# Efficacy of orally administered combination of moxidectin, sarolaner and pyrantel (Simparica Trio™) for the prevention of experimental *Angiostrongylus vasorum* infection in dogs

**DOI:** 10.1186/s13071-020-3948-z

**Published:** 2020-03-01

**Authors:** Csilla Becskei, Mirjan Thys, Padraig Doherty, Sean P. Mahabir

**Affiliations:** 1 Zoetis, Veterinary Medicine Research and Development, Mercuriusstraat 20, 1930 Zaventem, Belgium; 2 Charles River Laboratories, Pre-Clinical Services, Glenamoy, Mayo Ireland; 3 Zoetis Inc., Veterinary Medicine Research and Development, 333 Portage Street, Kalamazoo, MI 49007 USA

**Keywords:** Angiostrongylosis, Endectocide, French heartworm, Lungworm, Prevention

## Abstract

**Background:**

Infection with *Angiostrongylus vasorum* may cause severe clinical disease, even death in dogs, however, due to the often non-specific clinical signs, diagnosis is not always straightforward. Regular prophylactic treatment may offer a safe means to protect dogs against infection. The efficacy of a novel oral endectocide containing moxidectin, sarolaner and pyrantel was investigated for the prevention of angiostrongylosis in dogs in three placebo-controlled, randomized, masked studies. The initial study (Study 1) determined the efficacious dosage of moxidectin in the combination product by evaluating three different dose levels, and two follow-up studies (Studies 2 and 3) confirmed the efficacy of the selected moxidectin dose.

**Methods:**

Animals were infected orally with 200 infective third-stage larvae (L3) of *A. vasorum* and were treated 28 days later with the combination product or with placebo. Timing of dosing relative to infection allowed for efficacy to be evaluated against the immature adult (L5) stage. Dogs in Study 1 received treatments with oral tablets to deliver 3, 12 or 24 µg/kg moxidectin in combination with 2 mg/kg sarolaner and 5.0 mg/kg pyrantel (as pamoate salt) or placebo. In Studies 2 and 3, Simparica Trio™ tablets were administered to provide minimum dosages of 1.2 mg/kg sarolaner, 24 µg/kg moxidectin and 5.0 mg/kg pyrantel (as pamoate salt). Efficacy of the combination product was calculated as the percent reduction in adult worm counts at necropsy relative to placebo.

**Results:**

In Study 1, the 3, 12 and 24 µg/kg moxidectin dosage in the combination product provided 7.2%, 54.5% and 94.7% efficacy against the immature adult stages of *A. vasorum*, respectively. Studies 2 and 3 confirmed that the efficacy of 24 µg/kg moxidectin combined with 1.2 mg/kg sarolaner and 5 mg/kg pyrantel in Simparica Trio™ was ≥ 92.9%. All three studies established that a single oral administration of 24 µg/kg moxidectin in the combination product provided effective prophylactic treatment for angiostrongylosis, reduced L1 production and fecal excretion and minimized the tissue damage to the lungs.

**Conclusions:**

A single oral treatment of dogs with Simparica Trio™ providing moxidectin at a minimum dose of 24 µg/kg was efficacious in the prevention of angiostrongylosis.

## Background

Infections with nematodes that in their adult stages reside in the cardiopulmonary system may have serious impact on the health of their final host. *Angiostrongylus vasorum* (French heartworm or fox lungworm) and *Dirofilaria immitis* are two important species infecting dogs worldwide that may cause severe disease and, in some cases, even death [1, 2]. Both species have an indirect life-cycle.

*Angiostrongylus vasorum* is a metastrongyloid nematode that develops from the L1 stage to infective L3 in snails, slugs and frogs as intermediate hosts [2, 3]. The final hosts are canids [dogs, foxes (*Vulpes vulpes*, *Dusicyon vetulus*), coyotes (*Canis latrans*) etc.] that become infected by ingesting the intermediate hosts or taking up the L3 excreted by the intermediate hosts from the environment, e.g. by eating grass, chewing on sticks or drinking from puddles [2, 4]. Additionally, canids may be infected by ingesting paratenic hosts, such amphibians and birds [3, 5]. In the final host, L3 undergo two molts, the first one occurring in the abdominal lymph nodes within one week post-infection (pi) [6]. Following a second molt, the L5s reach the right ventricle and pulmonary arteries after about 10 days pi, where they develop into adults [6]. Eggs laid by the adult females hatch in the respiratory vessels and the L1 will be passed in the feces usually within 2 months pi. Infected dogs may excrete L1 for extended periods, but shedding may be intermittent or at a low level even in heavily infected dogs, making the diagnosis difficult [7].

Clinical diagnosis of canine angiostrongylosis is challenging because of the wide spectrum of clinical signs the infected dogs may present with and because subclinical infections also occur [2, 8]. Respiratory signs are most frequently reported, while infected dogs may also present with coagulopathies, cardiovascular, ophthalmological, gastrointestinal and neurological signs [8–10]. In rare cases sudden death may also occur [9, 11].

For the treatment of angiostrongylosis, topically administered moxidectin (in combination with imidacloprid; one or two monthly treatments) and oral milbemycin oxime (as monovalent or part of a combination product; once a week for four weeks) are licensed options in Europe [12–14]. Off label use of fenbendazole (daily oral treatment for several weeks) and ivermectin (weekly subcutaneous injections) has been also reported to be efficacious [9, 13, 15]. Due to the potentially severe disease in infected dogs and the difficulties in diagnosing angiostrongylosis, regular preventative treatment is a good option for dogs living in endemic regions. Licensed prophylactic medications belong to the macrocyclic lactones (topical moxidectin in combination with imidacloprid and oral milbemycin oxime in combination products) and have activity against the L4 and/or L5 stages [12].

Here, the results of three studies are presented that evaluated the efficacy of a novel chewable tablet containing sarolaner, moxidectin and pyrantel pamoate (Simparica Trio™, Zoetis, Parsipanny, NJ, USA) in the prevention of angiostrongylosis in experimentally infected dogs after a single oral administration. One of the studies aimed to determine the efficacious dose of moxidectin in the combination product; therefore, three different dose levels of moxidectin were tested simultaneously. The other two studies were conducted to confirm the efficacy of the selected moxidectin dose in the combination product.

## Methods

Three placebo-controlled, masked, randomized laboratory studies were conducted according to the World Association for the Advancement of Veterinary Parasitology (WAAVP) guidelines for evaluating the efficacy of anthelmintics for dogs and cats [16]. Study personnel who made assessments of efficacy or safety were masked to the treatment assignments of the dogs.

### Design

The study designs were based on the life-cycle of *A. vasorum* and previously published studies [14, 17, 18].

Study 1 was a dose determination study that aimed to identify the minimum effective dose of moxidectin in the combination product against the immature adult (L5) stage of *A. vasorum*. In this study, four groups of eight dogs each received oral treatment with either placebo or with the combination product that delivered an exact dose of 3, 12 or 24 µg/kg moxidectin. Two additional studies (Studies 2 and 3) confirmed the efficacy of the selected 24 µg/kg minimum moxidectin dose in the combination product against the L5 stage of *A. vasorum*. The study design of Studies 2 and 3 were identical, except for the origin of the *A. vasorum* isolates used for infection. In each of these studies, two groups of eight dogs each received oral treatment of placebo or the combination product.

In all three studies, dogs were infected orally with the L3 stage of *A. vasorum* on Day-28, received the study treatments on Day 0, at the time when the L3 stages were expected to have developed to immature adults (L5), and were necropsied once patency was confirmed in the placebo-treated dogs by fecal excretion of the L1 stage (28–42 days after treatment). The timing between the inoculation and the treatment of dogs (28 days) was determined based on the life-cycle of the parasite and previous study designs, in order to target the L5 stage at the time of treatment administration [14, 17, 18].

### Animals

Adult, purpose-bred Beagle dogs were used in all studies. Thirty-two dogs were enrolled in Study 1 in two cohorts (9 males and 7 females in cohort 1 and 8 males and 8 females in cohort 2). Sixteen dogs were used in both of Studies 2 and 3 (7 males and 9 females in each). The age of the dogs ranged between 11–45 months at enrolment and the body weights ranged between 10.2–18.5 kg at treatment administration. All dogs were in good health at enrolment as confirmed by a physical examination by a veterinarian and had undergone a wash-out period sufficient to ensure that no residual efficacy remained from any previously administered anthelmintics. All dogs were confirmed to be negative for *A. vasorum* before study start by three consecutive daily fecal examinations using the modified Baermann method. Dogs were housed in pairs until treatment administration and individually afterwards. Animal enclosures conformed to accepted animal welfare guidelines. Dogs received an appropriate maintenance ration of a commercial food for the duration of the study. Water was available *ad libitum*.

### Experimental infections

Animals received anti-emetic treatment (metoclopramide) and underwent general anesthesia (using acepromazine/buprenorphine/propofol combination) before experimental infections. Each dog was inoculated with 200 (± 10) viable *A. vasorum* infective L3 by gastric gavage. Animals were observed for 2 hours after inoculations for signs of vomiting. Animals that vomited in that time period were excluded from the study.

The parasite strains were collected as L1 stages from the feces of naturally infected dogs within approximately 1 year prior to study start. In Studies 1 and 3, the isolate originated from Italy, and in Study 2 from the UK. The L1 stages were inoculated into snails and were subsequently amplified by passage in donor dogs [17].

### Fecal examinations

Fecal samples were collected from all dogs on three consecutive days prior to inoculation (to ensure the negative status of the enrolled dogs) and post-treatment until L1 stages were recovered from the placebo-treated dogs (to ensure that patency had been reached). In Study 1, samples were collected on post-treatment days 23–28 for dogs enrolled in the first cohort and on days 23–35 in the second cohort. In Studies 2 and 3, the post-treatment samples were collected 32, 33 and 34 days after dosing. Each day, 10 grams feces was processed from each dog using the modified Baermann method to isolate and enumerate L1 larvae.

### Treatment

The studies followed a randomized complete block design (within cohort in Study 1), and block was based on age at enrolment. Dogs were ranked by age at enrolment and were randomly allocated to treatment groups and pens in each block.

In all studies, 28 days after the experimental infections dogs were dosed with either placebo tablets or with the combination product containing moxidectin, sarolaner and pyrantel. Placebo and active tablet presentations were similar in appearance in order to maintain masking. Body weights obtained within 2 days prior to treatment administrations were used for dose calculation. Food was withheld overnight prior to treatment and animals were not fed again until at least 4 hours after treatment. All doses were administered by hand pilling to ensure accurate dosing. Each dog was observed for several minutes after dosing for evidence that the dose was swallowed.

In Study 1, three formulations of the combination product were used that differed only in the moxidectin levels these contained (0.06, 0.24 or 0.48 mg/tablet) but not in the sarolaner (40 mg/tablet) and pyrantel (100 mg as pamoate salt/tablet) levels. Dogs in the three treatment groups received shaved and/or sanded combination product tablet(s) to deliver the exact moxidectin dose needed for each animal in each group i.e. 3, 12 or 24 µg/kg moxidectin. The combination product also delivered 2 mg/kg sarolaner and 5 mg/kg pyrantel (as pamoate salt) to the dogs. Dogs in the control group received an entire unshaved placebo tablet.

In Studies 2 and 3, Simparica Trio™ tablets were provided in four different sizes, such that a combination of tablets could be administered to ensure dogs were appropriately dosed to the minimum end of the label dose range of 1.2 mg/kg sarolaner + 24 µg/kg moxidectin + 5 mg/kg pyrantel (as pamoate salt) without under-dosing. Control dogs received the equivalent number of placebo tablets that were also provided in four different sizes.

### Necropsy and worm recovery

Dogs were humanely euthanized and necropsied in a predetermined random order. The dogs received sodium heparin intravenously (350 IU/kg) followed by intravenous injection of phenobarbital sodium at the label dosage. After euthanasia, the thorax was opened, and the extent of the lung lesions present on each of the six lobes (left cranial, left caudal, right cranial, right middle, right caudal and accessory) was recorded as the percentage of the consolidated lung areas. The severity of the lesions on each lobe was scored semi-quantitatively using a scale from 0 to 3 (0, normal; 1, mild; 2, moderate; and 3, severe) by the veterinarian. After the lung lesion scoring, reverse lung perfusion was performed on all euthanized animals as described before [17]. The collected blood and the perfusion solution was poured onto a fine sieve of ≤ 100 µm mesh size. The contents of the sieve were examined under a microscope to enumerate adult *A. vasorum*. Viability was checked by observing the movements of the worms (except for fragments). In the case of worm fragments, the total number of heads and the number of female and male tails were counted and recorded. All whole worms and worm fragments were counted and sexed (except the head fragments). After the perfusion, the lung tissue was finely sliced and flushed again. Any adults recovered from the lungs were counted. The sliced lung tissue was processed by the modified Baermann technique to isolate and count L1 stages.

### Statistical analysis

The experimental unit was the individual dog. The primary endpoint was the total adult worm counts recovered during necropsy. The total worm counts were transformed by the log_e_ (count + 1) transformation prior to analysis to stabilize the variance and normalize the data. Transformed counts were analyzed using a mixed linear model (SAS 9.3 and 9.4, Cary NC) that included the fixed effect of treatment, and the random effects of block and error. In Study 3, dogs were housed in several rooms, therefore the model included the random effects of room, block within room, and error. Testing was two-sided at the significance level α = 0.05.

Additionally, the number of the L1 stage larvae recovered from the lungs during necropsy and from the fecal examinations before necropsy were summarized. Percent reduction relative to placebo was calculated for the adult worm counts and the L1 counts from the lungs and the feces separately using geometric means (back-transformed least square means) based on the formula [(C − T)/C] × 100, where C is the mean worm count for the placebo group and T is the mean worm count for the treated group.

The mean percentage of the consolidated lung areas on each lobe and the sum of the individual lung lobe lesion severity scores were also calculated for each animal and were summarized by treatment group.

## Results

There were no mortalities and no treatment-related adverse reactions in any study. Infection was adequate in all studies with five or more worms found in at least six placebo-treated dogs in each study.

### Study 1

The placebo-treated dogs had adequate infection with geometric mean adult *A. vasorum* count of 53.8 (Table 1). Compared to placebo, the percent reduction in the adult worm counts was 7.2%, 54.5% and 94.7% in dogs that received 3 µg/kg, 12 µg/kg and 24 µg/kg moxidectin in the combination product, respectively. The adult worm counts were significantly lower in the 12 µg/kg and 24 µg/kg moxidectin groups compared to placebo (2.45 ≤ *t*_(21)_ ≤ 8.47, *P* ≤ 0.0234), but not in the 3 µg/kg group (*t*_(21)_ = 0.23, *P* = 0.8169). In the 24 µg/kg moxidectin group, significantly lower number of adult worms were recovered compared to the 3 µg/kg and the 12 µg/kg groups (6.02 ≤ *t*_(21)_ ≤ 8.23, *P* < 0.0001).

**Table 1:** Adult *Angiostrongylus vasorum* worm counts in Study 1: percent efficacy and statistical comparisons to placebo

Treatment	Moxidectin dose (µg/kg)^a^	Geometric mean worm count	% Efficacy	Test statistic
Placebo	0	53.8	–	–
Sarolaner + moxidectin + pyrantel	3	49.9	7.2	*t*_(21)_ = 0.23, *P* = 0.8169
Sarolaner + moxidectin + pyrantel	12	24.4	54.5	*t*_(21)_ = 2.45, *P* = 0.0234
Sarolaner + moxidectin + pyrantel	24	2.9	94.7	*t*_(21)_ = 8.47, *P* < 0.0001^b^

^a^The combination product-treated dogs received the exact moxidectin dose shown for each group, combined with 2 mg/kg sarolaner and 5 mg/kg pyrantel

^b^Adult worm counts significantly lower compared to the 3 µg/kg and the 12 µg/kg groups (6.02 ≤ *t*_(21)_ ≤ 8.23, *P* < 0.0001)

L1 stages were first recovered from fecal samples collected 24 days after treatment administration (52 days pi) in the placebo-treated dogs. The only group in which no L1 stages were found in the feces was the group that received 24 µg/kg moxidectin in the combination product (Table 2). The percent reduction in the fecal larvae counts compared to placebo was therefore 100% in that group.

**Table 2:** Study 1: *Angiostrongylus vasorum* L1 counts in the feces and lung, percent efficacy, mean percent consolidated lung lobe area and mean total lung lesion severity scores by treatment group

Treatment	Moxidectin dose (µg/kg)^a^	Fecal L1 counts^b^		Lung L1 counts		Lung lesions^c^	
		Geometric mean	% Efficacy	Geometric mean	% Efficacy	% Lung consolidation	Mean total severity score
Placebo	0	0.141	–	235.7	–	45.3	15.3
Sarolaner + moxidectin + pyrantel	3	0.894	0	762.2	0	42.0	14.3
Sarolaner + moxidectin + pyrantel	12	0.075	46.8	58.0	75.4	16.1	9.9
Sarolaner + moxidectin + pyrantel	24	0.000	100	0.0	100	3.1	5.6

^a^The combination product-treated dogs received the exact moxidectin dose shown for each group, combined with 2 mg/kg sarolaner and 5 mg/kg pyrantel

^b^Fecal L1 counts (per gram feces) were conducted daily on samples collected before necropsy for 5–12 days

^c^At necropsy the percentage of consolidation for each of the six lung lobes was assessed and the mean % consolidation was calculated. The severity of the lesions on each lobe was scored on a scale from 0 to 3 (the maximum possible total score was 18)

Following necropsy, no L1 stages were recovered from the lungs of dogs that received 24 µg/kg moxidectin in the combination product, while these were found in all other treatment groups (Table 2). Therefore, this dose provided 100% efficacy in the reduction in the lung larvae counts compared to placebo. In the placebo-treated group, on average 45.3% of the lung lobes showed macroscopic lesions with a mean total severity score of 15.3 out of a maximum score of 18, indicating severe organ damage (Table 2). In the combination product-treated dogs, the consolidated lung areas and the lesion severity scores decreased with increasing moxidectin dose. In the dogs receiving 24 µg/kg moxidectin, only 3.1% of the lung lobes showed lesions and the mean lung lesion severity score was 5.6.

### Studies 2 and 3

The placebo-treated dogs had adequate infection in both studies (Table 3). The percentage reduction in geometric mean adult worm count compared to the placebo-treated group was 94.0% in Study 2 and 92.9% in Study 3. In both studies, adult worm counts were significantly lower in the treated groups than in the respective placebo-treated groups (*t*_(8.83)_ = 5.95, *P* = 0.0002 and *t*_(7.71)_ = 6.83, *P* = 0.0002, respectively).

**Table 3:** Adult *Angiostrongylus vasorum* worm counts in Studies 2 and 3: percent efficacy and statistical comparisons to placebo

Study	Group^a^	Geometric mean worm count	% Efficacy	Test statistic
Study 2	Placebo	54.4	–	–
	Simparica Trio™	3.3	94.0	*t*_(8.83)_ = 5.95, *P* = 0.0002
Study 3	Placebo	94.8	–	–
	Simparica Trio™	6.8	92.9	*t*_(7.71)_ = 6.83, *P* = 0.0002

^a^The Simparica Trio™-treated dogs received 24 µg/kg moxidectin, 1.2 mg/kg sarolaner and 5 mg/kg pyrantel

L1 larvae were found in the feces of all placebo dogs on all three post-treatment sampling days in both studies, except for a single dog on a single sampling day (32 days after treatment) in Study 3. In the Simparica Trio™-treated dogs, no L1 larvae were found in the feces of any dogs in Study 2, and in Study 3 L1 larvae were only found in the feces of two out of the eight dogs. Compared to placebo, Simparica Trio™ treatment reduced fecal L1 counts by 100% and 98.7% in Study 2 and 3, respectively (Table 4).

**Table 4:** Studies 2 and 3: *Angiostrongylus vasorum* L1 counts in the feces and lung, percent efficacy, mean percent consolidated lung lobe area and mean total lung lesion severity scores by treatment group

Study	Group^a^	Fecal L1 counts^b^		Lung L1 counts		Lung lesions^c^	
		Geometric mean	% Efficacy	Geometric mean	% Efficacy	% Lung consolidation	Mean total severity score
Study 2	Placebo	2.349	–	157.4	–	54.5	11.3
	Simparica Trio™	0	100	0.0	100	1.9	2.7
Study 3	Placebo	3.793	–	336.2	–	59.0	12.0
	Simparica Trio™	0.050	98.7	1.9	99.4	1.2	0.9

^a^The Simparica Trio™-treated dogs received 24 µg/kg moxidectin, 1.2 mg/kg sarolaner and 5 mg/kg pyrantel

^b^Fecal L1 counts (per gram feces) were conducted daily on samples collected before necropsy for 3 days

^c^At necropsy the percentage of consolidation for each of the six lung lobes was assessed and the mean % consolidation was calculated. The severity of the lesions on each lobe was scored on a scale from 0 to 3 (the maximum possible total score was 18)

No L1 larvae were recovered from the lungs of the Simparica Trio™-treated dogs in Study 2, and compared to placebo, treatment reduced lung L1 counts by 99.4% in Study 3 (Table 4). In the placebo groups, 54.5% and 59.0% of the lung lobe areas showed *A. vasorum*-induced lesions, in Studies 2 and 3, respectively, while these were only found on 1.9% and 1.2% of the lung lobe areas in the Simparica Trio™-treated groups in Studies 2 and 3, respectively (Table 4). The severity of the lung lesions was also substantially reduced in the Simparica Trio™-treated groups.

## Discussion

The dose determination study (Study 1) established that 24 µg/kg was the minimum efficacious dose of moxidectin in the combination product to kill immature adults (L5) of *A. vasorum* and therefore, prevent the development of adult worms and reduce damage to the lungs of dogs after a single oral administration. The 24 µg/kg moxidectin dose provided 94.7% reduction in adult worm counts, completely prevented the development and excretion of L1 in the feces and markedly reduced the damage to the lung tissue in the treated animals. There was a dose response relationship of the efficacy of moxidectin against the L5 stages of *A. vasorum*. A dose of 3 µg/kg moxidectin in the combination product showed no efficacy and 12 µg/kg dose of moxidectin only reduced the adult worm counts by 54.5%. The two dose confirmation studies (Studies 2 and 3) demonstrated that Simparica Trio™ consistently provided ≥ 92.9% efficacy against the L5 stage of *A. vasorum* thus reducing the consequential lung tissue damage in dogs.

It is important to note that in the studies reported here, Simparica Trio™ provided high efficacy against *A. vasorum* infection after a single oral administration. Currently there are no licensed oral products available that provide efficacy after only a single treatment. In a study of similar design to the one reported here, treatment of dogs once or twice at a 28-day interval with milbemycin oxime at a dose of 0.5 mg/kg in combination with afoxolaner (Nexgard Spectra®) did not significantly reduce *A. vasorum* counts compared to placebo-treated dogs [19]. Three oral treatments of dogs at 28 days intervals with 0.51–0.96 mg/kg milbemycin oxime (in combination with afoxolaner), while significantly decreased the worm burden, did not achieve > 90% efficacy [19]. This combination product of milbemycin oxime only reached > 90% efficacy after four consecutive administrations at 28-day intervals when a trickle infection technique with repeated low dose (< 50 L3) infection of dogs every 2 weeks was used [20]. Simparica Trio™ may therefore provide an effective alternative to topical moxidectin (in combination with imidacloprid, Advocate®) when efficacy is expected after a single treatment administration [17].

Moxidectin at an oral dose of 3 µg/kg has been used for decades for the prevention of heartworm disease caused by *D. immitis* in dogs. This dose showed 100% efficacy against recent, susceptible field isolates, while at the 24 µg/kg dosage moxidectin provided improved efficacy against field isolates from the USA that were resistant to macrocyclic lactones [21]. In addition to the prophylactic efficacy against cardiovascular nematodes, the combination of moxidectin, sarolaner and pyrantel in Simparica Trio™ also provides effective treatment of flea, tick and gastrointestinal nematode infections [22–25]. This orally administered chewable tablet will therefore provide a convenient method for the pet owner to treat and control some of the most common internal and external parasites infecting or infesting dogs.

## Conclusions

These studies demonstrated the efficacy of a single oral dose of a new chewable tablet containing moxidectin, sarolaner and pyrantel (Simparica Trio™) against infection with immature adult (L5) *A. vasorum* in dogs.

## Data Availability

All data generated or analysed during this study are provided within the article.
